# 1*α*,25-Dihydroxyvitamin D_3_ (calcitriol) and its analogue, 19-nor-1*α*,25(OH)_2_D_2_, potentiate the effects of ionising radiation on human prostate cancer cells

**DOI:** 10.1038/sj.bjc.6601161

**Published:** 2003-08-12

**Authors:** N Dunlap, G G Schwartz, D Eads, S D Cramer, A B Sherk, V John, C Koumenis

**Affiliations:** 1 Department of Radiation Oncology, Comprehensive Cancer Center of Wake Forest University School of Medicine, Winston-Salem, NC 27157, USA; 2 Cancer Biology, Comprehensive Cancer Center of Wake Forest University School of Medicine, Winston-Salem, NC 27157, USA; 3 Department of Public Health Sciences, Comprehensive Cancer Center of Wake Forest University School of Medicine, Winston-Salem, NC 27157, USA; 4 Department of Urology, Comprehensive Cancer Center of Wake Forest University School of Medicine, Winston-Salem, NC 27157, USA

**Keywords:** Zemplar, vitamin D, radiosensitizer, synergistic

## Abstract

Radiotherapy with external beam radiation or brachytherapy is an established therapeutic modality for prostate cancer. Approximately 30% of patients with localised prostate cancer relapse at the irradiated site. Secondary effects of ionising radiation (IR), for example, bowel and bladder complications, are common. Thus, the search for biological response modifiers that could potentiate the therapeutic effects of radiation and limit the occurrence of serious side effects is an important task in prostate cancer therapy. 1*α*,25-Dihydroxyvitamin D_3_ (calcitriol), the active metabolite of vitamin D, and its analogues are under investigation for the treatment of several malignancies including prostate cancer. Here, we report that 1*α*,25-dihydroxyvitamin D_3_ and its less calcaemic analogue 19-nor-1*α*,25-(OH)_2_D_2_ (Zemplar®) act synergistically with IR to inhibit the growth of the human prostate cancer cells *in vitro*. 1*α*,25-dihydroxyvitamin D_3_ potentiated IR-induced apoptosis of LNCaP cells, and nanomolar doses of 1*α*,25-dihydroxyvitamin D_3_ and 19-nor-1*α*,25-(OH)_2_D_2_ showed synergistic inhibition of growth of LNCaP cells at radiobiologically relevant doses of IR (1–2 Gy). At higher doses of IR, the combination of 1*α*,25-dihydroxyvitamin D_3_ and IR or 19-nor-1*α*,25-(OH)_2_D_2_ and IR resulted in moderate antagonism. The synergistic effect at radiobiologically relevant doses of radiation suggests that a combination of 1*α*,25-dihydroxyvitamin D_3_ or 19-nor-1*α*,25-(OH)_2_D_2_ with IR could permit a reduction in the dose of radiation given clinically and thus potentially reduce treatment-related morbidity.

Adenocarcinoma of the prostate is the most commonly diagnosed nonskin cancer and the second leading cause of cancer death in men in the United States (Keely and Gomella, 1998; Garnik and Fair, 1998; Jemal *et al*, 2003). Most men will present with clinically localised disease but a significant minority will present with locally advanced disease. In patients with locally advanced disease (T3–4) the rates of local control with radiation therapy alone have been reported to range from 60 to 77% (Shipley *et al*, 1995). In a recent Phase III trial in men with locally advanced prostate cancer, the rate of local regional control in the radiation-alone arm was 83% (Bolla *et al*, 2002). Since this trial did not require prostate biopsies following treatment, the rate of loco-regional control is likely an overestimate.

It is clear that in men with locally advanced disease, optimal local control with radiation therapy has not been achieved. There is some evidence that higher radiation doses may improve local control, but higher doses lead to increased morbidity, especially rectal bleeding (Pollack *et al*, 2002). Whether used for cure or palliation, side effects of RT are common and include adverse effects on urinary, bowel and sexual function (Hamilton *et al*, 2001). Evidence also exists suggesting that the cells that survive the initial RT are the ones most likely to repopulate the irradiated area and metastasise (Fuks *et al*, 1991). Thus, agents or methods that could potentiate the effects of ionising radiation would be very desirable. Ideally, such agents would permit a reduction in the dose of radiation administered and thereby, a potential reduction in the incidence and/or severity of side effects.

The steroid hormone 1*α*,25-dihydroxyvitamin D_3_ (1*α*,25(OH)_2_D_3_), also known as calcitriol, the active metabolite of vitamin D, and its synthetic analogues are currently under intensive investigation for several malignancies, including prostate cancer (Campbell and Koeffler, 1997). Based on the observation that the descriptive epidemiology of prostate cancer resembles that of vitamin D insufficiency, Schwartz and Hulka (1990) proposed that vitamin D maintained the differentiated phenotype of prostate cells and that low levels of vitamin D increase the risk for prostate cancer. Many subsequent epidemiologic (Ahonen *et al*, 2000; Luscombe *et al*, 2001) and laboratory studies (Feldman *et al*, 2000) have supported this hypothesis. For example, 1*α*,25(OH)_2_D_3_ and its analogues exhibit significant growth-inhibitory, proapoptotic, anti-invasive and antimetastatic effects on prostate tumour cell lines *in* vitro and *in vivo* (Miller *et al*, 1992; Peehl *et al*, 1994; Schwartz *et al*, 1995,^1997^; Skowronski *et al*, 1995; Campbell *et al*, 1997; Asou *et al*, 1998; Lokeshwar *et al*, 1999; Blutt *et al*, 2000; Seol *et al*, 2000). These studies support the potential use of 1*α*,25(OH)_2_D_3_ and its analogues as therapeutic agents for prostate cancer (reviewed in Blutt and Weigel, 1999).

The principal limitation of 1,25(OH)_2_D_3_ administration is the risk of hypercalcaemia. Consequently, many synthetic analogues of 1,25(OH)_2_D_3_ have been developed with the goal of developing analogues that exhibit similar or enhanced growth-inhibitory and antimetastatic properties with reduced calcaemic effects (Schwartz and Hulka, 1990) (see also Hansen *et al* (2001) for a review). Prominent vitamin D analogues include EB1089 (Seocalcitol), which is currently in Phase III trials in Europe for hepatocellular carcinoma, 1-*α*-vitamin D_2_ and 19-nor-1α,25-(OH)_2_D_2_ (Zemplar), a 1,25(OH)_2_D_3_-analogue that is FDA-approved for the treatment of secondary hyperparathyroidism. Laboratory studies have demonstrated that the antiproliferative effects of 19-nor-1*α*,25-(OH)_2_D_2_ on prostate cancer cells are indistinguishable from those of 1α,25(OH)_2_D_3_ (Chen *et al*, 2000).

In addition to its use as monotherapy, calcitriol and analogues may prove useful in conjunction with other treatment modalities, notably, ionising radiation. Observations that support combining 1,25(OH)_2_D_3_ with ionising radiation include the following: (a) both IR and 1,25(OH)_2_D_3_ induce apoptosis in prostate carcinoma cells by apparently distinct pathways (Welsh *et al*, 1995; Billis *et al*, 1998; James *et al*, 1998; Pirianov *et al*, 1999; Ding and Fisher, 2001; McGuire *et al*, 2001; Sheard, 2001; Liu *et al*, 2002); (b) other agents that induce cellular differentiation, for example, phenylacetate, platelet-derived growth factor and retinoic acid, are known to enhance the cytotoxic effects of IR (Bill *et al*, 1992b; Miller *et al*, 1997; Hoffmann *et al*, 1999); (c) EB 1089 has been shown to enhance the radiation sensitivity of breast carcinoma cells (Sundaram and Gewirtz, 1999). These findings strongly suggest that 1,25(OH)_2_D_3_ may enhance the cytotoxic effects of IR on prostate cancer cells. In this report, we investigated the effects of combined treatments of 1*α*,25(OH)_2_D_3_ and 19-nor-1*α*,25-(OH)_2_D_2_ with IR on the apoptosis and growth of the human prostate cancer cell line LNCaP, as well as on the growth of a human primary prostatic cell strain obtained from patients who had undergone radical prostatectomy.

## MATERIALS AND METHODS

### Reagents

1*α*,25(OH)_2_D_3_ was purchased from Biomol, Inc. (Plymouth Meeting, PA, USA). 19-nor-1*α*,25(OH)_2_D_2_ was a generous gift from Abbott Laboratories (Chicago, IL, USA). Both were dissolved in 100% ethanol and kept at −80°C until use. Final concentration of EtOH in the media was 0.1% (V V^−1^).

### Cell culture

The LNCaP cell line was purchased from the American Type Culture Collection (Rockville, MD, USA) and maintained in RPMI 1640 supplemented with 10% foetal calf serum (FCS). Primary human epithelial cell cultures from histologically normal prostatic peripheral zones or prostate cancer were obtained from radical prostatectomies performed at Wake Forest University School of Medicine as previously described (Peehl and Stamey, 1986; Peehl *et al*, 1988; Barreto *et al*, 2000). Briefly, a small piece of tissue from each specimen is removed and minced. The tissue is digested with collagenase overnight. To remove the collagenase and the majority of the stromal cells, the tissue is rinsed and centrifuged. The collagenase-digested tissue is inoculated into a 60-mm tissue culture dish coated with collagen type I (Collagen Corporation, Palo Alto, CA, USA) and grown in medium PFMR-4A supplemented with growth factors and hormones (Peehl *et al*, 1988). Previous studies have demonstrated that prostatic stromal cells do not grow in the serum-free conditions used in this study, yet these conditions maintain the growth and differentiation of prostatic epithelial cells (Peehl and Stamey, 1986; Peehl *et al*, 1988). The cells that grew out from the tissue were aliquoted and stored in liquid nitrogen. The histology of each specimen was verified by inking and fixing the prostate after dissection and serially sectioning the marked area. The histology of sections immediately adjacent to the area of the dissection was reviewed and classified as histologically normal or cancerous by a trained pathologist. The frozen aliquots were thawed to produce secondary cultures, which were grown in medium MCDB 105 (Sigma, St Louis, MO, USA) supplemented with growth factors and hormones (Peehl and Stamey, 1986).

### Irradiation

LNCaP cells were plated in 24-well plates at densities of 5000 cells per well. Primary cells were plated onto 35 mm dishes at a cell density of 1 × 10^4^ dish^−1^. At the end of preincubation with 1α,25(OH)_2_D_3_ or 19-nor-1*α*,25(OH)_2_D_2_, cells were irradiated in a ^137^Caesium irradiator (Shepherd and Associates) at a dose rate of 472 rad min^−1^. Following IR, the cells were returned to the incubator and were allowed to grow for another 12–14 days, before assessment of viability as described below.

### Apoptosis assay

Cells were plated in 60 mm plastic dishes at a density of 2 × 10^5^ cells per plate 24 h prior to treatment. Following treatments, determination of the number of apoptotic cells was assayed. Cells with apoptotic morphology are determined by incubating cells with Bisbenzimide (Hoechst 33342) dye and propidium iodide (PI), both at a concentration of 2 *μ*g ml^−1^ for 10 min. Cells with apoptotic morphology (fragmented nuclei and PI-positive cells) were counted in each of four fields randomly selected in the dish and expressed as a ratio to total number of cells. Both attached and floating cells were counted. The results represent the average of three independent experiments (±s.e.m.).

### Cellular proliferation assays

Two types of cellular proliferation assays were employed. For assessing human prostatic cell strain proliferation, trypan-blue exclusion was used as a marker of viable cells 9–12 days after treatments. The WFU10Ca strain cells were plated onto 35 mm dishes and treated with 1*α*,25(OH)_2_D_3_ or ethanol, € EtOH). After 24 h later, cells were irradiated as described above. To assess the effects of 1*α*,25(OH)_2_D_3_ or 19-nor-1*α*,25(OH)_2_D_2_ on LNCaP cell proliferation, 5000 cells were plated onto 24-well plates and treated with 1*α*,25(OH)_2_D_3_ or 19-nor-1*α*,25(OH)_2_D_2_. Cells were irradiated 24 h later. After 10–12 days, cell viability was assessed by the MTT assay according to the manufacturer's protocol. A volume of 200 *μ*l aliquots from each reaction was transferred into a 96-well plate and absorbance values were measured with an automated plate reader (Molecular Dynamics, Sunnyvale, CA, USA). Each experiment was performed in triplicate.

### Isobologram analysis of combination treatments

The isobologram analysis was performed using the CalcuSyn software (Biosoft, Cambridge, UK). The analysis is based on the median-effect principle and the median effect equation by Chou (1974): *f*_a_/*f*_u_=(*D*/*D_m_*)^*m*^, where *D* is the dose of the drug; *D_m_* is the median-effect dose; *f*_a_ is the fraction affected by the dose, *f*_u_ the fraction unaffected by the dose, *f*_u_=1−*f*_a_; and *m* is an exponent signifying the sigmoidy (shape) of the dose–effect plot.

The Combination Index (CI) (Table 1

**Table 1:** Combination Index (CI) values and Dose Reduction Index (DRI) for combined treatments of vitamin D and IR (**A**) or Zemplar and IR (**B**)

Mutually nonexclusive CI for experimental values					Dose Reduction Index (DRI)	
	IR (Gy)	*f*_a_	CI (point #)	Degree of syn./antag.	Vitamin D	IR
(A) Vitamin D (nM)						
0.1	1	0.34	0.85 (1)	+	3.77	2.18
1	2	0.60	0.57 (2)	+++	9.46	2.36
10	4	0.74	0.76 (3)	++	6.70	1.89
100	4	0.75	2.43 (4)	−−−	0.79	1.96
(B) Zemplar (nM)					Zemplar	
0.1	1	0.45	1.27 (1)	−−	2.08	1.88
1	2	0.79	0.45 (2)	+++	10.81	3.05
10	4	0.89	0.54 (3)	+++	7.85	2.75
100	4	0.86	4.22 (4)	−−−−	0.39	2.23

CI values for each combination treatment at doses shown on the left were calculated with the CalCuSyn software as described in Materials and Methods. CI values correspond to points shown in the isobolograms depicted in Figure 3. The DRI for each drug:IR combination is a measure of how much (-fold) the dose of a drug or agent (e.g. IR) is synergistic combination may be reduced at a given effect level compared with the dose of each drug alone. The DRI is another mathematical interpretation of the CI, and CI=1/(DRI)_1_+1/(DRI)_2_. +++++, very strong agonism; −−−−− very strong antagonism

) for multiple drug combinations is calculated based on the equation and plot by Chou and Talalay:


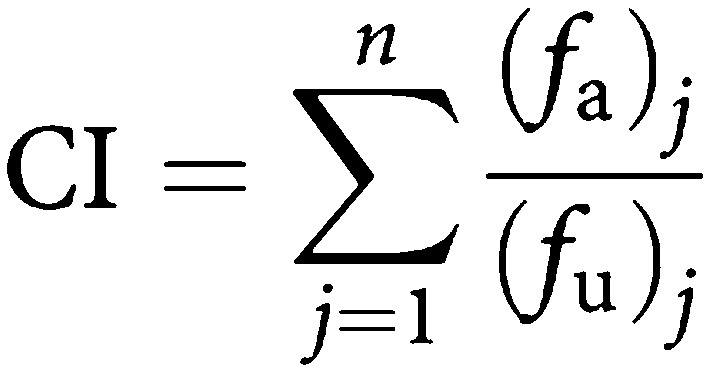


where *n*=number of drugs.

In the simplest form, a CI<1, =1 and >1 indicate synergism, additive effect and antagonism, respectively. Chou and Talalay (1984) also recommend a scale based on the value of CI and using symbols ranging from +++++ for very strong agonism to −−−−− for very strong antagonism. The CI values calculated from our experiments follow this scale.

## RESULTS

**Figure 1:**
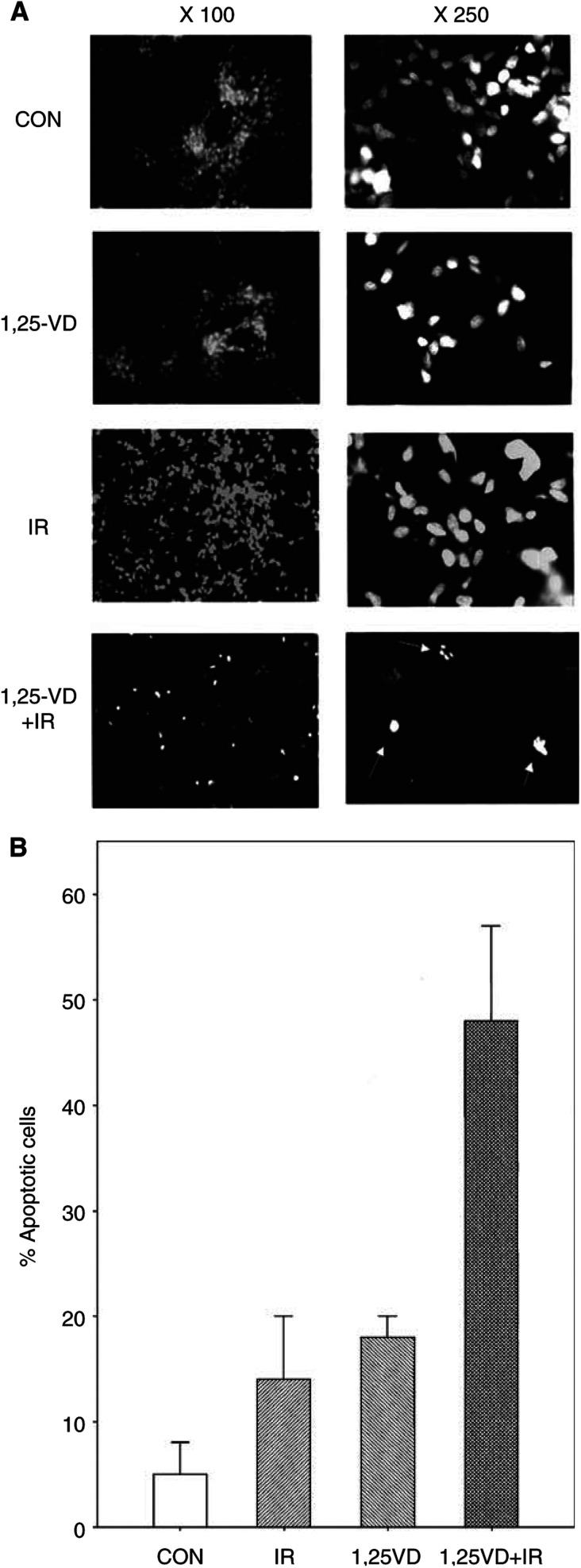
Pretreatment of LNCaP cells with 1*α*,25(OH)_2_D_3_ sensitises LNCaP cells to IR-induced apoptosis. (**A**) Photographs depict representative populations from the different treatment groups at × 100 and × 250 magnifications. Cells with apoptotic morphology appear with brighter stained nuclei (× 100 magnification). In the × 250 magnification, apoptotic cells are indicated by white arrows in the 1,25VD+IR treated group. (**B**) Cells were treated with ethanol (control) or 100 nM 1*α*,25(OH)_2_D_3_ for 48 h prior to treatment with 4 Gy IR and apoptotic cells were counted 12 h after IR. The apoptotic cells from four randomly selected fields were counted (between 50 and150 cells field^−1^). Three independent experiments were performed. Error bars represent±s.e. values.

To test whether 1*α*,25(OH)_2_D_3_ affects the apoptotic response of LNCaP cells to IR, cells were treated with vehicle (0.1% EtOH) or with 100 nM 1*α*,25(OH)_2_D_3_. After 48 h, the cells were either irradiated with 4 Gy IR or were mock-irradiated. Cells with apoptotic morphology (fragmented nuclei and PI-positive cells) were counted. Figure 1

shows photographs from representative populations at × 100 and × 250 magnifications. Treatment with 1*α*,25(OH)_2_D_3_ or IR alone did not induce substantial apoptosis of LNCaP cells, as is evident from the absence of cells with fragmented and/or condensed nuclei in this representative field. However, pretreatment with 1*α*,25(OH)_2_D_3_ for 48 h followed by IR resulted in a significant increase in the percentage of cells with apoptotic morphology (indicated by white arrows). Figure 1B shows the cumulative results obtained by counting cells with apoptotic *vs* normal morphology in four randomly selected fields from three independent experiments. Although 1*α*,25(OH)_2_D_3_ and IR induced only modest levels of apoptosis (13 and 17%, respectively) compared to basal levels (5%), pretreatment of LNCaP cells with 1*α*,25(OH)_2_D_3_ for 48 h prior to IR resulted in 48% apoptosis. These results indicate that pretreatment of LNCaP cells with 1*α*,25(OH)_2_D_3_ prior to IR sensitises these cells to IR-induced apoptosis.

**Figure 2:**
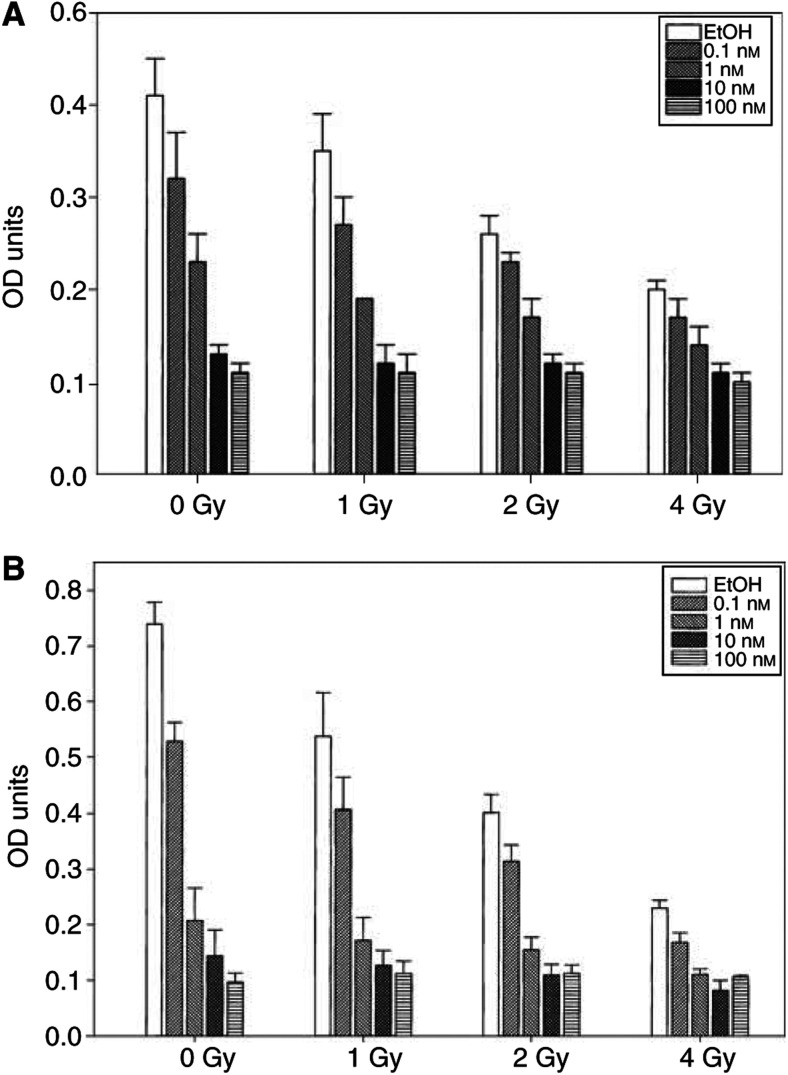
Dose–response effects of 1*α*,25(OH)_2_D_3_ (**A**) or 19-nor-1*α*,25(OH)_2_D_2_ (**B**) alone, or combined with IR on the growth of LNCaP prostate tumour cells. Cells were plated in medium and drugs were added 24 h later. Cells were irradiated 24 h following addition of 1*α*,25(OH)_2_D_3_ or 19-nor-1*α*,25(OH)_2_D_2_ and allowed to grow for an additional 7 days, at which time MTT assay was performed as described in Materials and Methods. Optical density (OD) values represent the average of four readings per experiment. Each experiment was performed three times. Error bars represent s.e. values.

**Figure 3:**
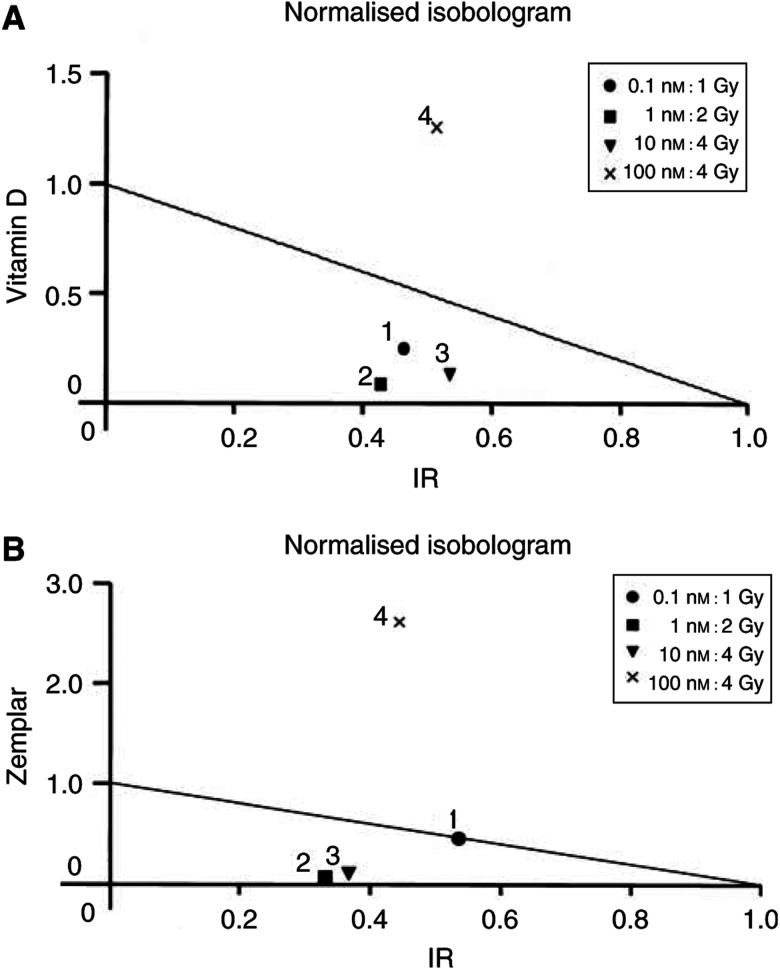
Isobologram plots for vitamin D and IR (**A**) or Zemplar and IR (**B**) combinations. Shown are normalised isobolograms indicating the equipotent combinations of the drugs at IC_50_ doses. As nonconstant ratios of drug : IR doses were used, the drug and IR concentrations on the isobol are normalised by the corresponding IC_50_ doses. Individual points refer to the specific dose ratios of combinations of drug : IR, indicated in the legend. If a combination data point for *f*_a_=0.5 falls on the diagonal, an additive effect is indicated; on the lower left, synergy is indicated; on the upper right, antagonism is indicated. For actual CI values and degrees of synergism or antagonism, see Tables 1 and 2.

While apoptotic assays can be useful in determining the short-term effects of agents on cells, they may not reflect the long-term effects of these agents on cellular proliferation. To investigate the effects of 1*α*,25(OH)_2_D_3_ and 19-nor-1*α*,25(OH)_2_D_2_ on long-term proliferation of prostate cells, we treated LNCaP cells with different doses of the hormones and IR, and assessed cell viability 8 days later using the MTT assay. As seen in Figure 2A and B

, both agents induced a dose-dependent decrease in the survival of LNCaP cells. An isobologram analysis of the data revealed that at lower doses of drug and IR, a significant but modest synergistic effect is observed (Figure 3A and B

). At increased concentrations of drug (100 nM) and IR (4 Gy) (point 4 in isobolograms), the two agents exert antagonistic effects on proliferation. These results suggest that the combination of low nanomolar doses of 1*α*,25(OH)_2_D_3_ or 19-nor-1*α*,25(OH)_2_D_2_ with low doses of IR may have beneficial effects *in vivo* for the treatment of prostate cancer.

**Figure 4:**
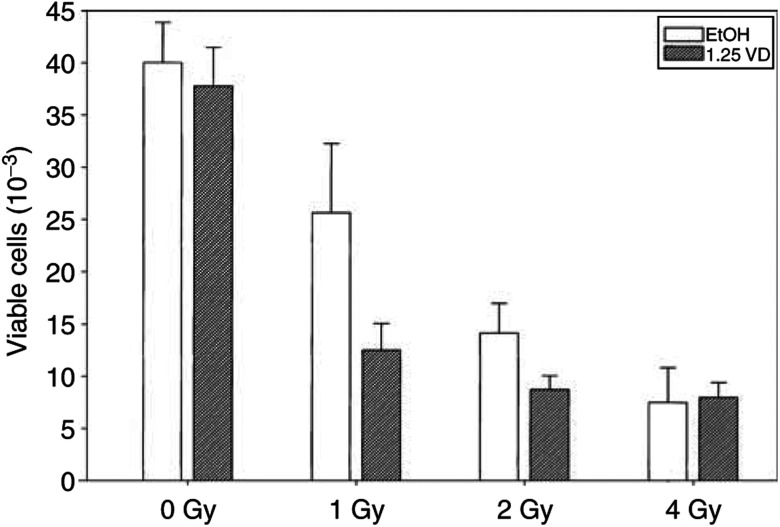
Effects of combined treatments of 1*α*,25(OH)_2_D_3_ and IR on primary prostate cells. Cells were isolated from patients who underwent radical prostatectomy at WFU as described in Materials and Methods. Cells were plated into 35 mm dishes and treated with 1 nM 1*α*,25(OH)_2_D_3_. After 24 h, cells were irradiated in a caesium irradiator with the indicated doses and returned to 37°C for growth for an additional 10–12 days. At the end of treatment, cells were trypsinised and counted. Control cells received only 0.1% ethanol. Experiments were performed in triplicate. The example shown is from the WFU10Ca, a cancer-derived primary strain. Similar results were obtained with two other cancer-derived and three normal prostate-derived strains.

We also wanted to test the effects of 1*α*,25(OH)_2_D_3_ on the response of primary prostate tumour cells to IR. Previously, it was shown that 1*α*,25(OH)_2_D_3_ inhibited the growth of primary prostatic cells in a dose-dependent manner (Peehl *et al*, 1994; Barreto *et al*, 2000). We first tested a range of doses of 1*α*,25(OH)_2_D_3_ for its ability to inhibit cell growth in a variety of normal and cancer-derived cell clones (data not shown). A dose of 1 nM had only a minimal effect on the growth of all strains tested. We next tested the effects of combined treatments of 1 nM 1*α*,25(OH)_2_D_3_ with increasing doses of IR. As shown in Figure 4

for a cancer-derived strain (WFU10Ca), IR treatments caused a dose-dependent decrease in cell proliferation. Pretreatment of these cells with 1 nM 1*α*,25(OH)_2_D_3_ caused a significant reduction in cell proliferation at 1 and 2 Gy, but it failed to further increase the inhibitory effect of 1*α*,25(OH)_2_D_3_ at a dose of 4 Gy. Therefore, similar to the results obtained with LNCaP cells, radiation and 1*α*,25(OH)_2_D_3_ act synergistically to inhibit cell proliferation at low doses of drug and IR.

A desired effect of radiation sensitisers/enhancers is a differential cytotoxic profile between tumour and normal cells. Thus, we also investigated the effects of the combined treatments of 1α,25(OH)_2_D_3_ and IR on the proliferative capacity of normal prostatic stromal cells, which are of fibroblastic origin and thus provide a good model for normal tissue injury. These cells were pretreated with various doses of 1*α*,25(OH)_2_D_3_ for 48 h, after which they were irradiated with doses of 1 and 2 Gy. Table 2

**Table 2:** Combination Index (CI) values, for combined treatments of vitamin D and IR in LNCap cells and normal prostatic stromal cells

	Mutually nonexclusive CI for experimental values				
	**Vitamin D (nM)**	**IR (Gy)**	***f*_a_**	**CI**	**Degree of syn.antag.**
LNCaP	0.1	2	0.45	0.77	++
	1	1	0.54	0.53	+++
	1	2	0.6	0.58	+++
	10	1	0.70	0.47	+++
Normal	0.1	2	0.27	1.135	−−
stromal cells	1	1	0.3	1.60	−−
	1	2	0.4	0.527	+++
	10	1	0.39	0.925	+

CI values for each combination treatment at doses shown on the left were calculated with the CalcuSyn software as described in Materials and methods. +++++, very strong agonism; −−−−− very strong antagonism.

shows the results of selected treatment combinations for these primary normal cells and the corresponding data for LNCaP cells. These data show that with the exception of the combined dose of 1 nM 1*α*,25(OH)_2_D_3_ and 2 Gy, which produced the highest levels of synergy for both the normal and LNCaP cells, the stromal cells were substantially more resistant to combined treatments than LNCaP cells. Furthermore, even at the synergistic combination of 1 nM 1*α*,25(OH)_2_D_3_ and 2 Gy, the overall inhibitory effect on stromal cell proliferation was lower than that observed for LnCaP cells, as evident by the affected fraction (*F*_a_) values (0.4 *vs* 0.6, respectively).

## DISCUSSION

1*α*,25(OH)_2_D_3_ and its analogues have shown synergistic activities with a variety of chemotherapeutic agents, including cisplatin and doxorubicin. In breast cancer cells, 1α,25(OH)_2_D_3_ and EB1089 were reported to enhance the effects of ionising radiation, although the lack of isobologram analysis in that study does not permit an assessment of a synergistic or additive effect (Sundaram and Gewirtz, 1999). Here, we demonstrate for the first time that in a prostate tumour cell line and primary tumour cells, 1*α*,25(OH)_2_D_3_ and 19-nor-1*α*,25(OH)_2_D_2_ act synergistically with IR to inhibit the growth of these tumour cells.

The synergistic effects of these agents with IR are manifested as an increase in apoptotic index of treated LNCaP cells, and as a decreased proliferation in longer-term assays in LNCaP and primary prostate cells. However, since the synergistic effect on apoptosis was observed with high doses of IR and 1*α*,25(OH)_2_D_3_, a combination that was antagonistic in longer-term assays, our results suggest that apoptosis is probably not involved in any synergistic long-term antiproliferative effects of these agents. One possible explanation for these results is the following: inhibition of proliferation by 1*α*,25(OH)_2_D_3_ and its analogues and IR involves multiple mechanisms, of which apoptosis is only one component. Terminal differentiation (or permanent cell cycle arrest) and mitotic (necrotic) death due to unrepaired DNA strand breaks also contribute substantially to decreased proliferation. It is possible that 1*α*,25(OH)_2_D_3_ and IR induce apoptosis via distinct mechanisms and therefore exhibit synergistic activation of apoptosis, but that differentiation and/or necrosis work by mechanisms that are mutually inhibited at higher drug or IR doses. This result is not surprising, since similar drug combinations (e.g., cisplatin and 1*α*,25(OH)_2_D_3_) also appear to produce antagonistic effects at high doses.

Although short-term apoptotic assays do not always demonstrate a good correlation with overall cell survival in *in vitro* assays, apoptosis may play a role *in vivo*. For example, increased apoptosis in a tumour as a result of treatment with a cytotoxic agent may contribute to reoxygenation of previously hypoxic tumour cells, and thus contribute to cytotoxicity of subsequent IR treatments (Brown and Wouters, 1999,^2001^). In LNCaP cells, the increased apoptotic index after a 48 h incubation suggests that pretreatment of cells with 1*α*,25(OH)_2_D_3_ lowers the apoptotic threshold of the cells to the subsequent stress of IR. A potential mechanism for this finding involves downregulation of the antiapoptotic protein bcl-2, which has been reported to occur as a result of treatment with 1*α*,25(OH)_2_D_3_ or its analogues (Welsh *et al*, 1995; Simboli-Campbell *et al*, 1997; Narvaez *et al*, 2001).

If an increase in apoptosis does not play a major role in the long-term antiproliferative synergistic interaction between 1*α*,25(OH)_2_D_3_ and its analogues, what other potential mechanisms may be involved in this process? First, calcitriol and its analogues induce cellular differentiation, a process in which a precursor cell acquires morphological and biochemical characteristics of specialised lineage (Studzinski *et al*, 1993). In the processes of tumorigenesis, cells reverse this process and become *less* differentiated (i.e., they dedifferentiate). Cells undergoing differentiation appear to be more sensitive to ionising radiation than cells that have a more undifferentiated phenotype. For instance, using a murine 3T3-T proadipocyte cell line, Bill *et al* have demonstrated that although both undifferentiated and differentiated cells sustain similar levels of DNA damage when irradiated, undifferentiated cells exhibit higher levels of DNA repair (Bill *et al*, 1992a, b). Similar mechanisms may explain the increased sensitivity of cells treated with the prodifferentiating agents phenylacetate and phenylbutyrate (Miller *et al*, 1997). Indeed, calcitriol has been shown in earlier studies to potentiate the cytotoxic effects of chemotherapeutic agents by inducing cell differentiation (Ravid *et al*, 1999). Second, treatment of the human prostate cells LNCaP and PC-3 with calcitriol results in cell cycle arrest, primarily at the G_1_/S interphase, while a G_2_/M arrest has also been reported (Eisman *et al*, 1989; Godyn *et al*, 1994; Zhuang and Burnstein, 1998). As shown by work with synchronised cell cultures, with the exception of the M phase of the cell cycle (where cells in culture are the most radiosensitive), the majority of cell lines show the greatest sensitivity to ionising radiation in late G_1_/early S phase and during the G_2_/M transition (Nias, 1988), and are the most radioresistant during early G_1_ and S phases. Therefore, it is reasonable to expect that pretreatment of prostate cancer cells with 1*α*,25(OH)_2_D_3_ and 19-nor-1*α*,25(OH)_2_D_2_ will increase their sensitivity to IR by inducing the entry of the majority of the cells in these two radiosensitive phases.

Regardless of the mechanism(s) of synergy, our data show that 1*α*,25(OH)_2_D_3_ and 19-nor-1*α*,25(OH)_2_D_2_ (which are both currently in clinical trials for the treatment of advanced prostate cancer) exhibit significant levels of synergy with one of the most widely used therapeutic modalities for prostate cancer, IR. Importantly, this potentiation occurs at low doses of both drug and IR, which fall well within the range of clinically relevant doses. For example, radiotherapy protocols for prostate cancer patients routinely employ multiple daily fractions of IR at 1.5–2 Gy per treatment for a total dose to the prostate between 64 and 72 Gy (Perez, 1997). As shown in our isobolograms, greatest synergism occurs at the IR doses of 2 Gy. Furthermore, the doses of 1*α*,25(OH)_2_D_3_ and 19-nor-1*α*,25(OH)_2_D_2_ that exhibit synergism are in the low nanomolar range. Intracellular levels of 1*α*,25(OH)_2_D_3_ in the prostate have not been characterised. However, it is now clear that these levels likely exceed by several fold the levels of 1*α*,25(OH)_2_D_3_ in the systemic circulation (picomolar levels (Platz *et al*, 2000)). This is because both normal and cancerous prostate cancer cells express 1-*α*-hydroxylase, which enables these cells to convert the prohormone 25-OHD_3_, which circulates at nanomolar concentrations, into 1*α*,25(OH)_2_D_3_, intraprostatically (Schwartz *et al*, 1998). Prostate cells that express 1-*α*-hydroxylase are growth inhibited by 25-OHD_3_ at nanomolar concentrations (Barreto *et al*, 2000). Therefore, both 1*α*,25(OH)_2_D_3_ and 19-nor-1*α*,25(OH)_2_D_2_ are capable of acting synergistically with IR at physiological doses, at least *in vitro*. Animal experiments will be required in order to validate these concepts *in vivo*.

Another conclusion of our study is that at higher doses of 1α,25(OH)_2_D_3_ and 19-nor-1*α*,25(OH)_2_D_2_ and IR, antagonism occurs. This finding is in agreement with similar studies using 1*α*,25(OH)_2_D_3_, cisplatin and carboplatin (Moffatt *et al*, 1999). Although the mechanism(s) for this antagonistic effect is unknown, it is well established that the synergy or antagonism between two drugs with different modes of action is largely dependent upon the drug concentrations and ratios. If these findings extend to *in vivo* models, they would suggest that careful dosage and patient monitoring must be employed if a combination of 1*α*,25(OH)_2_D_3_ or 19-nor-1*α*,25(OH)_2_D_2_ with IR is administered clinically.

The experiments with the primary cell cultures indicate that 1 nM 1*α*,25(OH)_2_D_3_ can also sensitise primary prostatic cells (both tumour and normal) to IR treatments. This finding supports the potential use of 1*α*,25(OH)_2_D_3_ and Zemplar as IR-response modifiers *in vivo*, since these cell strains retain most of the morphological and biochemical features of human prostatic tissue. Conversely, the ability of certain combinations of these agents to sensitise normal prostatic tissue to the effects of IR raises the possibility of increased radiation-induced adverse effects (i.e., prostatitis) in surrounding normal prostatic tissue. However, the data also suggest that at least *in vitro*, combinations of 1*α*,25(OH)_2_D_3_ and IR exist that might preferentially sensitise tumour but not normal prostate cells. Ultimately, such effective drug combinations will need to be established experimentally in either animal tumour models of prostate cancer before these agents are administered for therapeutic purposes in the clinic. Finally, it is possible that in a clinical setting, potential damage to normal tissue might be mitigated by brachytherapy in which radioactive ‘seeds’ are delivered locally to the tumour tissue, which would minimise the exposure of surrounding normal tissue to radiation.

The isobologram analysis and DRI of the effects of these agents on LNCaP cells also suggest that it may be possible to decrease the dose of IR and/or 1*α*,25(OH)_2_D_3_ and 19-nor-1*α*,25(OH)_2_D_2_ in combined modalities, without sacrificing efficacy. This finding has important clinical implications because the incidence of morbidity caused by RT increases with radiation dose (Perez, 1997). For instance, the DRI value of 2.4 for IR in the combination of 1*α*,25(OH)_2_D_3_ (1 nM) and IR (2 Gy) indicates that to achieve the same efficacy level in the absence of 1*α*,25(OH)_2_D_3_, one would have to give 2.4 times higher dose of IR. In other words, the presence of 1*α*,25(OH)_2_D_3_ allows for a 2.4 times lower dose of IR to obtain the same therapeutic effect. Conversely, the 9.5 DRI value for 1*α*,25(OH)_2_D_3_ indicates that at this combination of doses, the presence of IR allows for almost a 10-fold decrease in the level of 1*α*,25(OH)_2_D_3_ to achieve the same therapeutic effect. As noted above, one of the limiting factors of administration of 1*α*,25(OH)_2_D_3_ is hypercalcaemia. Our results show that by combining the two modalities it may be possible to reduce the levels of 1*α*,25(OH)_2_D_3_ or19-nor-1*α*,25(OH)_2_D_2_ without a significant compromise in efficacy, which would minimise the risk of hypercalcaemia.

In summary, we have shown that 1*α*,25(OH)_2_D_3_ and its less calcaemic analogue 19-nor-1*α*,25(OH)_2_D_2_ show synergistic effects when combined with radiation treatments. Both these drugs are approved for clinical use and both are presently under clinical investigation as monotherapy in prostate cancer. These findings have implications for limiting the disability (e.g., rectal and bladder injury) induced by radiation therapy. Future studies will focus on the *in vivo* interactions between these treatment modalities (using LNCaP xenograft tumour models) for the treatment of prostate cancer.

## References

[bib1] Ahonen MH, Tenkanen L, Teppo L, Hakama M, Tuohimaa P (2000) Prostate cancer risk and prediagnostic serum 25-hydroxyvitamin D levels (Finland). Cancer Causes Control 11: 847–852 11075874 10.1023/a:1008923802001

[bib2] Asou H, Koike M, Elstner E, Cambell M, Le J, Uskokovic MR, Kamada N, Koeffler HP (1998) 19-nor vitamin-D analogues: a new class of potent inhibitors of proliferation and inducers of differentiation of human myeloid leukemia cell lines. Blood 92: 2441–2449 9746784

[bib3] Barreto AM, Schwartz GG, Woodruff R, Cramer SD (2000) 25-Hydroxyvitamin D3, the prohormone of 1,25-dihydroxyvitamin D3, inhibits the proliferation of primary prostatic epithelial cells. Cancer Epidemiol Biomarkers Prev 9: 265–270 10750664

[bib4] Bill CA, Garrett KC, Harrell R, Tofilon PJ (1992a) Enhancement of radiation-induced cell killing and DNA double-strand breaks in a human tumor cell line using nanomolar concentrations of aclacinomycin A. Radiat Res 129: 315–321 1542719

[bib5] Bill CA, Grochan BM, Vrdoljak E, Mendoza EA, Tofilon PJ (1992b) Decreased repair of radiation-induced DNA double-strand breaks with cellular differentiation. Radiat Res 132: 254–258 1438708

[bib6] Billis W, Fuks Z, Kolesnick R (1998) Signaling in and regulation of ionizing radiation-induced apoptosis in endothelial cells. Recent Prog Horm Res 53: 85–92, discussion 93 9769704

[bib7] Blutt SE, Polek TC, Stewart LV, Kattan MW, Weigel NL (2000) A calcitriol analog, EB1089, inhibits the growth of LNCaP tumors in nude mice. Cancer Res 60: 779–782 10706079

[bib8] Blutt SE, Weigel NL (1999) Vitamin D and prostate cancer. Proc Soc Exp Biol Med 221: 89–98 10352118 10.1046/j.1525-1373.1999.d01-60.x

[bib9] Bolla M, Collette L, Blank L, Warde P, Dubois JB, Mirimanoff RO, Storme G, Bernier J, Kuten A, Sternberg C, Mattelaer J, Lopez Torecilla J, Pfeffer JR, Lino Cutajar C, Zurlo A, Pierart M (2002) Long-term results with immediate androgen suppression and external irradiation in patients with locally advanced prostate cancer (an EORTC study): a phase III randomised trial. Lancet 360: 103–106 12126818 10.1016/s0140-6736(02)09408-4

[bib10] Brown JM, Wouters BG (1999) Apoptosis, p53, and tumor cell sensitivity to anticancer agents. Cancer Res 59: 1391–1399 10197600

[bib11] Brown JM, Wouters BG (2001) Apoptosis: mediator or mode of cell killing by anticancer agents? Drug Resist Update 4: 135–136 10.1054/drup.2001.0193 11512523

[bib12] Campbell MJ, Elstner E, Holden S, Uskokovic M, Koeffler HP (1997) Inhibition of proliferation of prostate cancer cells by a 19-nor-hexafluoride vitamin D3 analog involves the induction of p21waf1, p27kip1 and E-cadherin. J Mol Endocrinol 19: 15–27 9278857 10.1677/jme.0.0190015

[bib13] Campbell MJ, Koeffler HP (1997) Toward therapeutic intervention of cancer by vitamin D compounds. J Natl Cancer Inst 89: 182–185 9016994 10.1093/jnci/89.3.182

[bib14] Chen TC, Schwartz GG, Burnstein KL, Lokeshwar BL, Holick M.F (2000) The *in vitro* evaluation of 25-hydroxyvitamin D3 and 19-nor-1alpha,25-dihydroxyvitamin D2 as therapeutic agents for prostate cancer. Clin Cancer Res 6: 901–908 10741714

[bib15] Chou T (1974) Relationships between inhibition constants and fractional inhibition in enzyme-catalyzed reactions with different numbers of reactants, different reaction mechanisms, and different types and mechanisms of inhibition. Mol Pharmacol 10: 235–247 4212316

[bib16] Chou TC, Talalay P (1984) Quantitative analysis of dose–effect relationships: the combined effects of multiple drugs or enzyme inhibitors. Adv Enzyme Regul 22: 27–55 6382953 10.1016/0065-2571(84)90007-4

[bib19] Ding HF, Fisher DE (2001) p53, caspase 8, and regulation of apoptosis after ionizing radiation. J Pediatr Hematol Oncol 23: 185–188 11305724 10.1097/00043426-200103000-00014

[bib20] Eisman JA, Koga M, Sutherland RL, Barkla DH, Tutton PJ (1989) 1,25-Dihydroxyvitamin D3 and the regulation of human cancer cell replication. Proc Soc Exp Biol Med 191: 221–226 2740355 10.3181/00379727-191-42912

[bib21] Feldman D, Zhao XY, Krishnan AV (2000) Vitamin D and prostate cancer. Endocrinology 141: 5–9 10614617 10.1210/endo.141.1.7341

[bib22] Fuks Z, Leibel SA, Wallner KE, Begg CB, Fair WR, Anderson LL, Hilaris BS, Whitmore WF (1991) The effect of local control on metastatic dissemination in carcinoma of the prostate: long-term results in patients treated with 125I implantation. Int J Radiat Oncol Biol Phys 21: 537–547 1869452 10.1016/0360-3016(91)90668-t

[bib23] Garnick MB, Fair WR (1998) Combating prostate cancer. Sci Am 279: 74–83 9828467 10.1038/scientificamerican1298-74

[bib24] Godyn JJ, Xu H, Zhang F, Kolla S, Studzinski GP (1994) A dual block to cell cycle progression in HL60 cells exposed to analogs of vitamin D3. Cell Prolif 27: 37–46 10465025 10.1111/j.1365-2184.1994.tb01404.x

[bib26] Hamilton AS, Stanford JL, Gilliland FD, Albertsen PC, Stephenson RA, Hoffman RM, Eley JW, Harlan LC, Potosky AL (2001) Health outcomes after external-beam radiation therapy for clinically localised prostate cancer: results from the Prostate Cancer Outcomes Study. J Clin Oncol 19: 2517–2526 11331331 10.1200/JCO.2001.19.9.2517

[bib27] Hansen CM, Binderup L, Hamberg KJ, Carlberg C (2001) Vitamin D and cancer: effects of 1,25(OH)2D3 and its analogues on growth control and tumorigenesis. Front Biosci 6: D820–D848 11438443 10.2741/hansen

[bib28] Hoffmann W, Blase MA, Santo-Hoeltje L, Herskind C, Bamberg M, Rodemann HP (1999) Radiation sensitivity of human squamous cell carcinoma cells *in vitro* is modulated by all-*trans* and 13-*cis*-retinoic acid in combination with interferon-alpha. Int J Radiat Oncol Biol Phys 45: 991–998 10571207 10.1016/s0360-3016(99)00298-9

[bib29] James SY, Mercer E, Brady M, Binderup L, Colston KW (1998) EB1089, a synthetic analogue of vitamin D, induces apoptosis in breast cancer cells *in vivo* and *in vitro*. Br J Pharmacol 125: 953–962 9846632 10.1038/sj.bjp.0702103 PMC1565659

[bib30] Jemal A, Murray T, Samuels A, Ghafoor A, Ward E, Thun MJ (2003) Cancer statistics, 2003. CA Cancer J Clin 53: 5–26 12568441 10.3322/canjclin.53.1.5

[bib32] Keely FX, Gomella LG (1998) Epidemiology of Prostate Cancer. In Prostate Cancer, Ernstoff MS, Heany JA, Peschel RE (eds) pp 2–14. London: Blackwell Sciences

[bib33] Liu G, Oettel K, Ripple G, Staab MJ, Horvath D, Alberti D, Arzoomanian R, Marnocha R, Bruskewitz R, Mazess R, Bishop C, Bhattacharya A, Bailey H, Wilding G (2002) Phase I trial of 1alpha-hydroxyvitamin D(2) in patients with hormone refractory prostate cancer. Clin Cancer Res 8: 2820–2827 12231522

[bib34] Lokeshwar BL, Schwartz GG, Selzer MG, Burnstein KL, Zhuang SH, Block NL, Binderup L (1999) Inhibition of prostate cancer metastasis *in vivo*: a comparison of 1,25-dihydroxyvitamin D (calcitriol) and EB1089. Cancer Epidemiol Biomarkers Prev 8: 241–248 10090302

[bib35] Luscombe CJ, Fryer AA, French ME, Liu S, Saxby MF, Jones PW, Strange RC (2001) Exposure to ultraviolet radiation: association with susceptibility and age at presentation with prostate cancer. Lancet 358: 641–642 11530156 10.1016/S0140-6736(01)05788-9

[bib36] McGuire TF, Trump DL, Johnson CS (2001) Vitamin D(3)-induced apoptosis of murine squamous cell carcinoma cells. Selective induction of caspase-dependent MEK cleavage and up-regulation of MEKK-1. J Biol Chem 276: 26365–26373 11331275 10.1074/jbc.M010101200

[bib37] Miller AC, Whittaker T, Thibault A, Samid D (1997) Modulation of radiation response of human tumour cells by the differentiation inducers, phenylacetate and phenylbutyrate. Int J Radiat Biol 72: 211–218 9269314 10.1080/095530097143437

[bib38] Miller GJ, Stapleton GE, Ferrara JA, Lucia MS, Pfister S, Hedlund TE, Upadhya P (1992) The human prostatic carcinoma cell line LNCaP expresses biologically active, specific receptors for 1 alpha,25-dihydroxyvitamin D3. Cancer Res 52: 515–520 1370648

[bib39] Moffatt KA, Johannes WU, Miller GJ (1999) 1Alpha,25dihydroxyvitamin D3 and platinum drugs act synergistically to inhibit the growth of prostate cancer cell lines. Clin Cancer Res 5: 695–703 10100724

[bib40] Narvaez CJ, Zinser G, Welsh J (2001) Functions of 1alpha,25-dihydroxyvitamin D(3) in mammary gland: from normal development to breast cancer. Steroids 66: 301–308 11179738 10.1016/s0039-128x(00)00202-6

[bib41] Nias A (1988) Clinical Radiobiology. New York: Churchill Livingstone

[bib42] Peehl DM, Skowronski RJ, Leung GK, Wong ST, Stamey TA, Feldman D (1994) Antiproliferative effects of 1,25-dihydroxyvitamin D3 on primary cultures of human prostatic cells. Cancer Res 54: 805–810 7508338

[bib43] Peehl DM, Stamey TA (1986) Serum-free growth of adult human prostatic epithelial cells. *In Vitro* Cell Dev Biol 22: 82–90 2419302 10.1007/BF02623537

[bib44] Peehl DM, Wong ST, Stamey TA (1988) Clonal growth characteristics of adult human prostatic epithelial cells. *In Vitro* Cell Dev Biol 24: 530–536 3391931 10.1007/BF02629087

[bib45] Perez CA (1997) Prostate. In Principles and Practices in Radiation Oncology, Perez CA, Brady LW (eds) pp 1583–1694. Philadelphia: Lippincot-Raven

[bib46] Pirianov G, Danielsson C, Carlberg C, James SY, Colston KW (1999) Potentiation by vitamin D analogues of TNFalpha and ceramide-induced apoptosis in MCF-7 cells is associated with activation of cytosolic phospholipase A2. Cell Death Differ 6: 890–901 10510471 10.1038/sj.cdd.4400563

[bib47] Platz EA, Hankinson SE, Hollis BW, Colditz GA, Hunter DJ, Speizer FE, Giovannucci E (2000) Plasma 1,25-dihydroxy- and 25-hydroxyvitamin D and adenomatous polyps of the distal colorectum. Cancer Epidemiol Biomarkers Prev 9: 1059–1065 11045788

[bib48] Pollack A, Zagars GK, Starkschall G, Antolak JA, Lee JJ, Huang E, von Eschenbach AC, Kuban DA, Rosen I (2002) Prostate cancer radiation dose response: results of the M. D. Anderson phase III randomized trial. Int J Radiat Oncol Biol Phys 53: 1097–1105 12128107 10.1016/s0360-3016(02)02829-8

[bib49] Ravid A, Rocker D, Machlenkin A, Rotem C, Hochman A, Kessler-Icekson G, Liberman UA, Koren R (1999) 1,25-Dihydroxyvitamin D3 enhances the susceptibility of breast cancer cells to doxorubicin-induced oxidative damage. Cancer Res 59: 862–867 10029076

[bib51] Schwartz GG, Hill CC, Oeler TA, Becich MJ, Bahnson RR (1995) 1,25-Dihydroxy-16-ene-23-yne-vitamin D3 and prostate cancer cell proliferation *in vivo*. Urology 46: 365–369 7660511 10.1016/S0090-4295(99)80221-0

[bib52] Schwartz GG, Hulka BS (1990) Is vitamin D deficiency a risk factor for prostate cancer? (Hypothesis). Anticancer Res 10: 1307–1311 2241107

[bib53] Schwartz GG, Wang MH, Zang M, Singh RK, Siegal GP (1997) 1 alpha,25-Dihydroxyvitamin D (calcitriol) inhibits the invasiveness of human prostate cancer cells. Cancer Epidemiol Biomarkers Prev 6: 727–732 9298581

[bib54] Schwartz GG, Whitlatch LW, Chen TC, Lokeshwar B, Holick LMF (1998) Human prostate cells synthesize 1,25-dihydroxyvitamin D3 from 25-hydroxyvitamin D3. Cancer Epidemiol Biomarkers Prev 7: 391–395 9610788

[bib55] Seol JG, Park WH, Kim ES, Jung CW, Binderup L, Koeffler HP, Kim BK, Lee YY (2000) Effect of a novel vitamin D3 analog, EB1089, on G1 cell cycle regulatory proteins in HL-60 cells. Int J Oncol 16: 315–320 10639575

[bib56] Sheard MA (2001) Ionizing radiation as a response-enhancing agent for CD95-mediated apoptosis. Int J Cancer 96: 213–220 11474495 10.1002/ijc.1020

[bib57] Shipley WU, Verhey LJ, Munzenrider JE, Suit HD, Urie MM, McManus PL, Young RH, Shipley JW, Zietman AL, Biggs PJ, Heney NM, Goitein M (1995) Advanced prostate cancer: the results of a randomized comparative trial of high dose irradiation boosting with conformal protons compared with conventional dose irradiation using photons alone. Int J Radiat Oncol Biol Phys 32: 3–12 7721636 10.1016/0360-3016(95)00063-5

[bib58] Simboli-Campbell M, Narvaez CJ, van Weelden K, Tenniswood M, Welsh J (1997) Comparative effects of 1,25(OH)2D3 and EB1089 on cell cycle kinetics and apoptosis in MCF-7 breast cancer cells. Breast Cancer Res Treat 42: 31–41 9116316 10.1023/a:1005772432465

[bib59] Skowronski RJ, Peehl DM, Feldman D (1995) Actions of vitamin D3, analogs on human prostate cancer cell lines: comparison with 1,25-dihydroxyvitamin D3. Endocrinology 136: 20–26 7530193 10.1210/endo.136.1.7530193

[bib60] Studzinski GP, McLane JA, Uskokovic MR (1993) Signaling pathways for vitamin D-induced differentiation: implications for therapy of proliferative and neoplastic diseases. Crit Rev Eukaryot Gene Expr 3: 279–312 8286848

[bib61] Sundaram S, Gewirtz DA (1999) The vitamin D3 analog EB 1089 enhances the response of human breast tumor cells to radiation. Radiat Res 152: 479–486 10521924

[bib62] Welsh J, Simboli-Campbell M, Narvaez CJ, Tenniswood M (1995) Role of apoptosis in the growth inhibitory effects of vitamin D in MCF-7 cells. Adv Exp Med Biol 375: 45–52 7645427 10.1007/978-1-4899-0949-7_4

[bib63] Zhuang SH, Burnstein KL (1998) Antiproliferative effect of 1alpha,25-dihydroxyvitamin D3 in human prostate cancer cell line LNCaP involves reduction of cyclin-dependent kinase 2 activity and persistent G1 accumulation. Endocrinology 139: 1197–1207 9492054 10.1210/endo.139.3.5770

